# Attitude of Dental Students Toward Rubber Dam Application: A Cross-Sectional Study

**DOI:** 10.7759/cureus.60503

**Published:** 2024-05-17

**Authors:** Nagham E Saleem, Abrar Y Baitalmal, Reham M Alsamman, Shahinaz N Sembawa

**Affiliations:** 1 College of Dental Medicine, Umm Al-Qura University, Makkah, SAU; 2 Division of Operative Dentistry, Department of Restorative Dentistry, College of Dental Medicine, Umm Al-Qura University, Makkah, SAU; 3 Division of Dental Public Health, Department of Preventive Dentistry, College of Dental Medicine, Umm Al-Qura University, Makkah, SAU

**Keywords:** operative dentistry, dental students, attitude, moisture control, field isolation, rubber dam

## Abstract

Introduction

The rubber dam (RD) system is globally recognized as an indispensable component of dental treatments, ensuring the highest standards of care. The use of RD isolation profoundly impacts the clinical aspects of dental procedures. The aim of this study was to assess the attitudes of dental students toward the use of RD, with an emphasis on evaluating the factors influencing its use among students at Umm Al-Qura University in Makkah city, Kingdom of Saudi Arabia.

Methods

An online questionnaire was distributed to a population sample of 203 undergraduate dental students in their clinical years at Umm Al-Qura University. Questions covered various aspects, including the frequency of RD use, perceptions of its effectiveness, and prospective intentions for its future use, as well as factors influencing these attitudes. Statistical analysis was performed using SPSS software Version 26 (IBM Corp., Armonk, NY, USA). In addition, the chi-square test and Fisher-Freeman-Halton exact test were used for the comparison of the data at a significance level of p<0.05.

Results

The questionnaire was completed by 191 participants, with a response rate of 95.5%. Of the students, 189 (99%) used RD during operative procedures for adhesive restorations. Furthermore, 157 (82.2%) students agreed that proper isolation cannot be achieved without RD application. All the advantages of RD application were recognized by 165 (85.9%) of the students. Notably, there was a significant association between the factors limiting the RD application and the students’ academic year (p<0.05), with senior students reporting fewer limitations. Responses indicated that 163 (85.3%) of the students received satisfactory training for RD and 165 (86.4%) students had a high intention to continue using RD following graduation.

Conclusion

Within the limitations of this study, it can be concluded that the results presented a positive attitude toward RD use. However, there is a continued necessity to enhance education and advocate consistent RD application for improved patient care and clinical outcomes.

## Introduction

The rubber dam (RD) system is globally recognized as an indispensable component of dental treatments, ensuring the highest standards of care [1,2]. The use of RD isolation profoundly impacts the clinical aspects of dental procedures. By creating a controlled environment, RD enhances precision during treatment, minimizing contamination of the operative field and ensuring optimal visibility for the dentist [3-6]. This controlled isolation not only aids in infection control but also prevents the accidental ingestion or aspiration of instruments, irritants, and materials, thus significantly reducing the risk of procedural complications [3-6].

RD not only creates an aseptic operative field and controls moisture, crucial for maintaining the mechanical integrity and longevity of restorative treatments [7], but also ensures proper bonding of dental materials, thereby enhancing the durability and success of such restorations [7]. Failure to use RD is considered substandard care from a medicolegal perspective, potentially exposing dentists and patients to unnecessary risks and legal repercussions [8].

RD isolation is commonly employed in two major areas of dentistry: endodontics and operative dentistry. Textbooks and experts in both specialties recommend the use of RD routinely [1,2]. With the advancement of modern dentistry and the increasing focus on conservative and adhesive techniques, the use of RD has become essential for achieving optimal treatment outcomes [9,10]. Despite the well-established importance of its use, underuse among general practitioners remains prevalent [10-13]. Thus, modern dental education is adopting the philosophy of safe and high quality of care, emphasizing the consistent application of RD.

Dental schools in Saudi Arabia have structured their curricula to enhance students' knowledge and skills in RD application. However, a lingering question persists regarding whether students will embrace this practice post-graduation. Conducting a questionnaire-based survey among students can interpret the attitudes of the future dental workforce and shed light on the inherent challenges associated with RD use, thereby facilitating the development of an enhanced educational strategy. Therefore, the aim of this study is to evaluate dental students' attitudes toward RD use, with a particular focus on factors influencing their experiences and intentions regarding its application post-graduation.

## Materials and methods

A population sample of all clinical-level dental students at the College of Dental Medicine at Umm Al-Qura University, Makkah, Kingdom of Saudi Arabia, were invited to participate in the study. Anonymous online questionnaire was distributed to students in their fourth, fifth, sixth, and internship years. Approval for the study was obtained from the Biomedical Research Ethics Committee of Umm Al-Qura University (approval No. HAPO-02-K-012). The questionnaire was designed using Google Forms and was made available to 203 participants between January 13, 2023, and February 28, 2023.

The questionnaire (Appendix 1), comprising 17 questions, was formulated based on modifications of previously validated questionnaires [14,15]. It was structured into the following three sections:

Consent for participation: This section obtained participants' voluntary consent to partake in the study. Participants were assured of the confidentiality of their responses.

Demographic information: Participants were asked to provide information on gender and academic year.

Attitudes toward RD use: This section, comprising 15 close-ended and multiple-choice questions, explored participants' attitudes and perspectives regarding the use of RD. Questions covered various aspects, including the frequency of RD use, perceptions of its effectiveness, and prospective intentions for its future use, as well as factors influencing these attitudes.

Statistical analysis was performed using SPSS software Version 29 (IBM Corp., Armonk, NY, USA). In addition to descriptive statistical methods, the chi-square test and Fisher-Freeman-Halton exact test were used for the comparison of the data. Results were evaluated at a significance level of p < 0.05.

## Results

A total of 191 completed questionnaires were received, representing a response rate of 95.5%. The gender distribution among the respondents was as follows: 99 (51.8%) were female and 92 (48.2%) were male. The sample comprised fourth-year students (46, 24.1%), fifth-year students (44, 23%), sixth-year students (50, 26.2%), and interns (51, 26.7%). In terms of RD use, 189 (99%) students reported using RD in adult patients, and 163 (85.3%) students reported using RD in children. A portion of the students (114, 59.7%) checked patients for latex allergies before applying RD. Nearly, all students (189, 99%) used RD during operative procedures for adhesive restorations. Furthermore, 157 (82.2%) out of 191 students agreed that proper isolation cannot be achieved without RD application, with a significant association (p=0.005) with the students' academic level (Table 1).

**Table 1:** Use of rubber dam among dental students in association with academic year. *Pearson chi-square test; **Fisher-Freeman-Halton exact test; ***Significant difference between academic years at p <0.05.

Academic years					Total, N=191	Test value	P-value
Fourth year		Fifth year	Sixth year	Interns			
46 (24.1%)		44 (23%)	50 (26.2%)	51 (26.7%)			
Do you use a rubber dam in adults?							
Yes	45 (23.6%)	44 (23%)	50 (26.2%)	50 (26.2%)	189 (99%)	2.1**	0.86
No	1 (0.5%)	0	0	1 (0.5%)	2 (1%)		
Do you use a rubber dam in a child?							
Yes	34 (17.8%)	42 (22%)	49 (25.7%)	38 (19.9%)	163 (85.3%)	19.6*	<0.001***
No	12 (6.3%)	2 (1%)	1 (0.5%)	13 (6.8%)	28 (14.7%)		
Do you use a rubber dam during operative procedures for adhesive restorations, for example, composite restorations?							
Yes	45 (23.6%)	44 (23%)	50 (26.2%)	50 (26.2%)	189 (99%)	2.1**	0.86
No	1 (0.5%)	0	0	1 (0.5%)	2 (1%)		
Do you agree that proper isolation cannot be achieved for the restoration of operative procedures without rubber dam?							
Yes	38 (19.9%)	29 (15.2%)	47 (24.6%)	43 (22.5%)	157 (82.2%)	12.9*	0.005***
No	8 (4.2%)	15 (7.9%)	3 (1.6%)	8 (4.2%)	34 (17.8%)		
Do you frequently use the rubber dam for the establishment of core and after root canal treatment?							
Yes	38 (19.9%)	38 (19.9%)	40 (20.9%)	41(21.5%)	157 (82.2%)	0.8*	0.85
No	8 (4.2%)	6 (3.1%)	10 (5.2%)	10 (5.2%)	34 (17.8%)		
Do you ask your patients whether they have an allergy to latex before rubber dam use?							
Yes	31 (16.2%)	26 (13.6%)	26 (13.6%)	31 (16.2%)	114 (59.7%)	2.4*	0.495
No	15 (7.9%)	18 (9.4%)	24 (12.6%)	20 (10.5%)	77 (40.3%)		

Most of the students (169, 88.5%) agreed that RD is essential for procedures involving both jaws, with a significant association with academic year, as sixth-year students presented the highest agreement compared to others (p=0.01). The location of the teeth showed an effect on the frequency of using the RD, with the mandibular posterior teeth scoring the highest frequency (181, 94.8%). Moreover, the application of RD in the mandibular anterior teeth varied according to the academic year (p<0.05). Additionally, the academic level of the student significantly affected their decision on the methods of isolation, whether to use RD or partial isolation (p=0.003), and the timing of applying RD in the restorative and endodontic treatments (p<0.05) (Table 2).

**Table 2:** Attitude of dental students toward RD use in various clinical situations by academic year. *Pearson chi-square test; ** Fisher-Freeman-Halton exact test; ***Significant difference between academic years at p <0.05.

Academic years					Total, N=191	Test value	P-value
Fourth year		Fifth year	Sixth year	Interns			
46 (24.1%)		44 (23%)	50 (26.2%)	51(26.7%)			
Do you frequently use the rubber dam for isolation in? Select all that apply							
Maxillary anterior	40 (20.9%)	35 (18.3%)	47 (24.6%)	45 (23.6%)	167 (87.4%)	4.5*	0.21
Maxillary posterior	36 (18.8%)	36 (18.8%)	46 (24.1%)	45 (23.6%)	163 (85.3%)	4.4*	0.22
Mandibular anterior	37 (19.4%)	33 (17.3%)	46 (24.1%)	47 (24.6%)	163 (85.3%)	8.3*	0.04***
Mandibular posterior	42 (22%)	41 (21.5%)	50 (26.2%)	48 (25.1%)	181 (94.8%)	4.8**	0.18
RD is more necessary while working in the...?							
Maxilla	1 (0.5%)	0	0	0	1 (0.5%)	13.2**	0.01***
Mandible	3 (1.6%)	9 (4.7%)	1 (0.5%)	8 (4.2%)	21 (11%)		
Both jaws	42 (22%)	35 (18.3%)	49 (25.7%)	43 (22.5%)	169 (88.5%)		
Do you frequently use the rubber dam for isolation or use cotton roll with suction (partial isolation)?							
Rubber dam	31(16.2%)	18 (9.4%)	34 (17.8%)	20 (10.5%)	103 (53.9%)	16.9**	0.003***
Cotton roll with suction (partial isolation)	0	0	0	1 (0.5%)	1 (0.5%)		
Depends on the case	15 (7.9%)	26 (13.6%)	16 (8.4%)	30 (15.7%)	87 (45.5%)		
During which stage of restoration do you use a rubber dam?							
Following anesthesia before cavity preparation	44 (23%)	42 (22%)	49 (25.7%)	41 (21.5%)	176 (92.1%)	10.8**	0.007***
After cavity preparation before placement of restoration	2 (1%)	2 (1%)	1 (0.5%)	10 (5.2%)	15 (7.9%)		
During which stage of endodontic treatment do you use the rubber dam?							
Following anesthesia	37 (19.4%)	41 (21.5%)	46 (24.1%)	46 (24.1%)	170 (89%)	18.2**	0.02***
During access cavity preparation	2 (1%)	1 (0.5%)	3 (1.6%)	3 (1.6%)	9 (4.7%)		
Following identification of root canal orifices	7 (3.7%)	0	0	2 (1%)	9 (4.7%)		
During root canal shaping	0	1 (0.5%)	0	0	1(0.5%)		
During root canal filling	0	1 (0.5%)	1 (0.5%)	0	2 (1%)		

**Figure 1:**
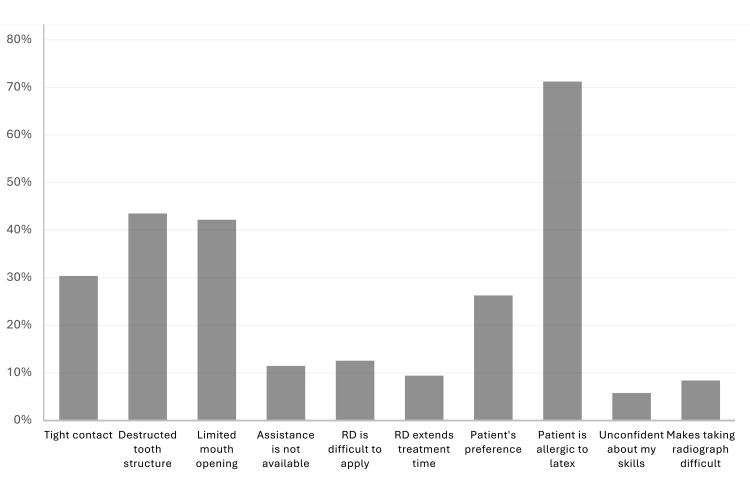
Elements affecting the use of RD. The distribution of the factors limiting the RD application among clinical-level dental students (N= 191) highlights the most common trends. RD, rubber dam

Regarding the advantages offered by RD, 164 (85.9%) students agreed that RD provides an aseptic working area, prevents swallowing or aspiration of particles, eases access to cavity preparation and restoration, and enhances the longevity of restorations (Table 3). Despite these advantages, 136 (71.2%) students reported patients' latex allergies as the primary factor for not using RD, followed by destructed tooth structure (83, 43.7%), limited mouth opening (81, 42.4%), and tight contact (58, 30.4%) (Figure 1). Some of these factors presented a significant association with the academic year of the student (p<0.05) as the reported limitations decreased with the advancement in academic level (Table 3).

**Table 3:** Opinions of clinical-level dental students on the application of RD according to academic year. *Pearson chi-square test; **Fisher-Freeman-Halton exact test; ***Significant difference between academic years at p <0.05. RD, rubber dam

Academic years					Total, N=191	Test value	P-value
Fourth year		Fifth year	Sixth year	Interns			
46 (24.1%)		44 (23%)	50 (26.2%)	51(26.7%)			
In your opinion, the greatest advantage offered by the RD is/are?							
Aseptic working area	3 (1.6%)	2 (1%)	3 (1.6%)	1 (0.5%)	9 (4.7%)	8.25**	0.77
Prevention of swallowing or aspirating particles	4 (2.1%)	3 (1.6%)	3 (1.6%)	1 (0.5%)	11 (5.8%)		
Easy access to cavity preparation and restoration	2 (1%)	1 (0.5%)	1 (0.5%)	0	4 (2.1%)		
Greater longevity of restoration	1 (0.5%)	1 (0.5%)	0	1 (0.5%)	3 (1.6%)		
All of the above	36 (18.8%)	37 (19.4%)	43 (22.5%)	48 (25.1%)	164 (85.9%)		
Why wouldn’t you place a RD during treatment? Select all that apply.							
RD is difficult to apply	12 (6.3%)	6 (3.1%)	3 (1.6%)	3 (1.6%)	24 (12.6%)	11.7*	0.008***
Tight contact	23 (12%)	19 (9.9%)	10 (5.2%)	6 (3.1%)	58 (30.4%)	22.7*	<0.001***
Destructed tooth structure	16 (8.4%)	19 (9.9%)	21 (11%)	27 (14.1%)	83 (43.5%)	3.3*	0.38
Limited mouth opening	24 (12.6%)	17 (8.9%)	21 (11%)	19 (9.9%)	81 (42.4%)	2.6*	0.46
Need assistance and it is not available	9 (4.7%)	7 (3.7%)	4 (2.1%)	2 (1%)	22 (11.5%)	7.25*	0.05
RD extends treatment time	7 (3.7%)	6 (3.1%)	3 (1.6%)	2 (1%)	18 (9.4%)	5.1**	0.16
Patient’s preference	14 (7.3%)	14 (7.3%)	12 (6.3%)	10 (5.2%)	50 (26.2%)	2.4*	0.47
Patient is allergic to latex	28 (14.7%)	36 (18.8%)	34 (17.8%)	38 (19.9%)	136 (71.2%)	5.3*	0.15
Unconfident about my skills in applying RD	6 (3.1%)	4 (2.1%)	1 (0.5%)	0	11 (5.8%)	9.4**	0.009***
RD makes taking radiograph difficult	8 (4.2%)	4 (2.1%)	3 (1.6%)	1 (0.5%)	16 (8.4%)	7.4**	0.05

The majority of students (163, 85.3%) reported receiving adequate and satisfactory training in terms of RD application. Additionally, a significant proportion of the respondents (165, 86.4%) expressed their intention to use RD for all procedures following graduation, with a statistical difference observed among academic years (p=0.02) (Table 4).

**Table 4:** Dental students’ satisfaction with training and their intended use of RD post-graduation. *Pearson chi-square test; **Fisher-Freeman-Halton exact test; ***Significant difference between academic years at p <0.05. RD, rubber dam

Academic years					Total, N=191	Test value	P-value
Fourth year		Fifth year	Sixth year	Interns			
46 (24.1%)		44 (23%)	50 (26.2%)	51(26.7%)			
Do you think you have been given adequate and satisfactory training regarding RD?							
Yes	35 (18.3%)	38 (19.9%)	46 (24.1%)	44 (23%)	163 (85.3%)	5*	0.38
No	11 (5.8%)	6 (3.1%)	4 (2.1%)	7 (3.7%)	28 (14.7%)		
Following graduation?							
I intend to use RD during all procedures	36 (18.8%)	41 (21.5%)	47 (24.6%)	41 (21.5%)	165 (86.4%)	16.3**	0.02***
I intend to use RD only during restorative procedures	4 (2.1%)	0	1 (0.5%)	0	5 (2.6%)		
I intend to use RD only during root canal treatment	3 (1.6%)	1 (0.5%)	2 (1%)	8 (4.2%)	14 (7.3%)		
I intend to use other forms of isolation rather than RD	3 (1.6%)	2 (1%)	0	2 (1%)	7 (3.7%)		

## Discussion

The present study aimed to assess the attitudes of dental students toward the use of RDs and the factors influencing their experience, as well as their intentions regarding RD application following graduation. Our findings present a positive attitude among dental students toward the use of RDs. Specifically, 189 (99%) of respondents reported using RDs in adult patients, particularly during adhesive restorative procedures. In contrast, the application of RDs in pediatric patients varied across academic years, with lower use observed. Notably, our study reported a higher prevalence of RD use in pediatric patients (163 out of 191, 85.3%) compared to the findings reported by Tanalp et al. [14].

The dental students at Umm Al-Qura University recognized the importance of RD application, with 169 (88.5%) of respondents agreeing on its necessity for both maxillary and mandibular jaws. Interestingly, this finding contrasts with several studies where students predominantly considered RD more essential for procedures involving the mandible [14-16]. Additionally, the mandibular posterior teeth were the most frequent location for using RD. This could potentially be attributed to factors such as saliva pooling, as well as movement of the tongue and cheek during treatment. While most students favored RD over partial isolation, half of the respondents indicated that their choice of isolation method and moisture control would vary depending on the specific case. Interestingly, a higher percentage of interns expressed this viewpoint, suggesting that as the students progress through their academic journey, they develop a more critical approach to treatment planning.

Regarding the advantages of RD application, 164 (85.9%) of respondents acknowledged all its benefits, including providing an aseptic working area, preventing swallowing or aspiration of particles, easing access to cavity preparation and restoration, and enhancing the longevity of restorations. This is in disagreement with findings from a study [15] conducted at a private dental school in Jeddah, Saudi Arabia, where undergraduate dental students identified the facilitation of an aseptic work area as the utmost advantage provided by RD, with only 17 (15.4%) out of 110 participants recognizing all the mentioned advantages.

In clinical settings, 114 (59.7%) students inquired about latex allergies prior to RD use, indicating the level of awareness regarding the protocol of RD use. This finding is consistent with results reported in several studies conducted across various dental schools in Saudi Arabia [15-18]. Latex allergy presents an absolute contraindication for RD application, and neglecting this fact can potentially compromise patient safety. These findings underscore the importance of addressing potential areas for improvement in this aspect of dental practice.

Regarding factors affecting the use of RD, our study identified patient sensitivity to latex as the main reason for not using RD, followed by destructed tooth structure, limited mouth opening, and tight contact. Additionally, fourth-year students reported difficulties in application and lack of confidence, with these concerns diminishing as the students progress through their academic years and training. Most participants expressed satisfaction with their training and showed a strong inclination toward using RD in all indicated procedures following graduation. These findings align with those of several previous studies [15,16,18], indicating that effective teaching and clinical training at dental schools play a crucial role in boosting students' confidence and shaping their future clinical practice.

The group of dental students under study began their RD training during the third academic year in a preclinical setting. This training included theoretical lectures, live demonstrations, and practical hands-on sessions. As they transitioned to the clinical stage, their skills developed through case-based learning that they gained from treating a variety of patients. Different strategies for teaching RD application can be suggested to enhance the learning outcomes and assist dental students in navigating diverse clinical situations, especially those deemed challenging. Research has highlighted educational videos as an effective tool in teaching clinical procedures among dental and medical students [19,20]. These videos expose students to different levels of complexity and encourage critical thinking in handling challenging clinical situations. Additionally, dental manikins can be modified to simulate real-life scenarios, addressing the reported limitations such as tight contacts, destructed tooth structure, and limited mouth opening. Exposing the students to multiple teaching approaches will improve their competency and boost their level of confidence in adapting the practice of RD application.

The current study has several limitations, including a relatively small sample size and a specific location. Our investigations were confined to one university in Saudi Arabia (Umm Al-Qura University, Makkah); thus, the outcomes reported may lack representativeness for dental students nationwide. Future studies should aim to include larger sample sizes and incorporate other major universities in Saudi Arabia to enhance the generalizability of findings.

## Conclusions

Within the limitations of this study, it can be concluded that undergraduate dental students at Umm Al-Qura University presented a positive attitude toward RD application. Challenges such as patient latex sensitivity and clinical and anatomical variations were identified as barriers to its widespread adoption. However, as students advance through their academic years and gain clinical experience, these concerns diminish, emphasizing the essential role of effective teaching in enhancing confidence and promoting the consistent use of RD in dental practice. Our findings highlight reinforcing awareness and training on the best practices of RD application, where dental schools and professional organizations can contribute to improved patient care and clinical outcomes.

## Appendices

Appendix 1: The online questionnaire used for assessment.

1. Gender

·       Male

·       Female

2. Academic year

·       Fourth-year dental student

·       Fifth-year dental student

·       Sixth-year dental student

·       Intern

3. Do you use a rubber dam in adults?

·       Yes

·       No

4. Do you use a rubber dam in a child?

·       Yes

·       No

5. Do you use a rubber dam during operative procedures for adhesive restorations, for example composite restorations?

·       Yes

·       No

6. Do you agree that proper isolation cannot be achieved for restoration of operative procedures without rubber dam?

·       Yes

·       No

7.  Do you frequently use the rubber dam for establishment of core and post after root canal treatment?

·       Yes

·       No

8. Do you ask your patients whether they have an allergy to latex before rubber dam use?

·       Yes

·       No

9. Do you frequently use the rubber dam for isolation in? Select all that apply.

·       Maxillary anterior

·       Maxillary posterior

·       Mandibular anterior

·       Mandibular posterior

10. Rubber dam is more necessary while working in the...?

·       Maxilla

·       Mandible

·       Both jaws

11. Do you frequently use the rubber dam for isolation, or use cotton roll with suction (partial isolation)?

·       Rubber dam.

·       Cotton roll with suction (partial isolation)

·       Depends on the case.

12. During which stage of restoration do you use a rubber dam?

·       Following anesthesia before cavity preparation

·       After cavity preparation before placement of restoration

13. During which stage of endodontic treatment do you use the rubber dam?

·       Following anesthesia

·       During access cavity preparation

·       Following identification of root canal orifices

·       During root canal shaping

·       During root canal filling

14. In your opinion, the greatest advantage offered by the rubber dam is/are?

·       Aseptic working area.

·       Prevention of swallowing or aspirating particles or instruments.

·       Easy access to cavity preparation and restoration

·       Restorations placed under rubber dam have a greater longevity than those placed without

·       All above

15. Why wouldn’t you place a rubber dam during treatment? Select all apply.

·       Rubber dam is difficult to apply

·       Due to tight contact

·       Destructed tooth structure

·       Limited opening of patient mouth

·       Need assistance and it is not available

·       Rubber dam extends treatment procedures time

·       Patient’s preference

·       Patients have allergy to latex

·       I don’t feel confident about my skills in applying rubber dam

·        It makes taking radiograph difficult

16. Do you think you have been given adequate and satisfactory training regarding rubber dam?

·       Yes

·       No

17. Following graduation:

·       I intend to use the rubber dam during all procedures indicated

·       I intend to use it only during restorative procedures

·       I intend to use it only during root canal treatment

·       I intend to use other forms of isolation rather than rubber dam
